# Characterization of a new potent and long-lasting single chain peptide agonist of RXFP1 in cells and in vivo translational models

**DOI:** 10.1038/s41598-022-24716-2

**Published:** 2022-11-28

**Authors:** Stephane Illiano, Bruno Poirier, Claire Minoletti, Olivier Pasquier, Laurence Riva, Xavier Chenede, Isabelle Menguy, Michel Guillotel, Philippe Prigent, Stéphane Le Claire, Florence Gillot, Gilbert Thill, François Lo Presti, Alain Corbier, Jean-Christophe Le Bail, Patrick Grailhe, Edith Monteagudo, Raffaele Ingenito, Elisabetta Bianchi, Christophe Philippo, Olivier Duclos, Sergio Mallart, Ross Bathgate, Philip Janiak

**Affiliations:** 1 Cardio-Vascular and Metabolism, Sanofi R&D, 1 avenue Pierre Brossolette, 91385 Chilly-Mazarin, France; 2 Integrated Drug Discovery, Sanofi R&D, 1 avenue Pierre Brossolette, 91385 Chilly-Mazarin, France; 3 DMPK France, Sanofi R&D, 1 avenue Pierre Brossolette, 91385 Chilly-Mazarin, France; 4 Translational Science, Sanofi R&D, 1 avenue Pierre Brossolette, 91385 Chilly-Mazarin, France; 5 Peptides and Small Molecules R&D Department, ≠DMPK, and Structural Biology IRBM Spa, Via Pontina Km 30 600, 00 071 Pomezia, RM Italy; 6 Florey Institute of Neuroscience and Mental Health and Department of Biochemistry and Pharmacology, The University of Melbourne, Parkville, VIC 3052 Australia; 7 IRSN, B.P. 17, 92262 Fontenay-Aux-Roses Cedex, France; 8 Present Address: Structural biology and chemistry, Institut Pasteur, PARIS, France

**Keywords:** Peptide nucleic acid oligo, Heart failure

## Abstract

Despite beneficial effects in acute heart failure, the full therapeutic potential of recombinant relaxin-2 has been hampered by its short half-life and the need for intravenous administration limiting its use to intensive care units. A multiparametric optimization of the relaxin B-chain led to the identification of single chain lipidated peptide agonists of RXFP1 like SA10SC-RLX with subcutaneous bioavailability and extended half-life. SA10SC-RLX has sub nanomolar activity on cells expressing human RXFP1 and molecular modeling associated with the study of different RXFP1 mutants was used to decipher the mechanism of SA10SC-RLX interaction with RXFP1. Telemetry was performed in rat where SA10SC-RLX was able to engage RXFP1 after subcutaneous administration without tachyphylaxis after repeated dosing. Renal blood flow was then used as a translational model to evaluate RXFP1 activation. SA10SC-RLX increased renal blood flow and decreased renal vascular resistance in rats as reported for relaxin in humans. In conclusion, SA10SC-RLX mimics relaxin activity in in vitro and in vivo models of acute RXFP1 engagement. SA10SC-RLX represents a new class of long-lasting RXFP1 agonist, suitable for once daily subcutaneous administration in patients and potentially paving the way to new treatments for chronic fibrotic and cardiovascular diseases.

## Introduction

Relaxin is a heterodimeric 2-chain peptide hormone structurally related to insulin. The RLN-2 gene in humans and the RLN-1 gene from other mammals encode relaxin-2 and relaxin-1, respectively, which are commonly referred to as relaxin. RXFP1 is the main relaxin receptor and is expressed in various tissues (heart, kidney, lung, skin, liver, blood vessels, and brain). Relaxin also activates RXFP2 but there is no evidence of physiological functions of RXFP2 activation by relaxin in humans. Relaxin has multiple complex binding interactions with RXFP1 which direct the N-terminal LDLa module of the receptor together with a linker domain to act potentially as a tethered ligand to promote signaling^1^. RXFP1 is a Gs protein-coupled receptor and the main signaling pathway triggered following activation is cyclic adenosine monophosphate (cAMP). Other signaling pathways have been reported including Gi activation in vascular cells. Over time, RXFP1 recruits coupling to G(alpha)(i3), causing additional cAMP accumulation via a G(alpha)(i3)-G beta gamma-phosphoinositide 3-kinase (PI3K)-protein kinase C (PKC) zeta pathway^2^.

The axis relaxin/RXFP1 plays a key role in pregnancy and reproduction where it contributes to the maintenance of myometrial quiescence and softening, the hypertrophy of the cervix, and the development of the mammary glands and papilla^3^. In addition, relaxin is involved in the hemodynamic changes that occur during pregnancy, including the increase in cardiac output and renal blood flow, reduction in peripheral arterial resistance and elevation in arterial compliance^4^. At the vasculature level relaxin causes vasorelaxation through nitric oxide and prostaglandin I2 production, promotes angiogenesis via the upregulation of vascular endothelial growth factor and prevents inflammation^5^. At the kidney level relaxin increases renal blood flow and glomerular filtration in healthy subjects via activation of RXFP1^6,7^. Relaxin also displays anti-fibrotic properties in various preclinical models of hepatic, pulmonary, renal and cardiovascular fibrosis while RXFP1 knockout mice develop perivascular pulmonary fibrosis with time^8^. In addition, relaxin has been reported to limit cardiac or kidney ischemia/reperfusion injury^9^.

These features highlight the therapeutic potential of RXFP1 activation for the treatment of heart failure, pulmonary arterial hypertension, liver and kidney fibrosis, scleroderma, or idiopathic pulmonary fibrosis. Consistently the hemodynamic effects of relaxin have been recapitulated in healthy volunteers or patients with chronic heart failure and have prompted its investigations in larger clinical trials in patients with acute heart failure, where a 2-day infusion of relaxin was shown initially to improve renal and cardiac function resulting in reduced pulmonary congestion and mortality at 6 months^10^. However, these results on cardiovascular mortality were not confirmed in a second phase III trial (RELAX-AHF-2), suggesting that a 2-day treatment was too short to ensure a significant and durable recovery from acute decompensated heart failure. The very short half-life of relaxin, in human, has limited its use to short-term treatment in hospital settings and it is not suited for chronic treatment. This highlighted the need for the development of long-lasting relaxin mimetics. Attempts to address this issue were recently reported using recombinant relaxin bearing natural or non-natural amino acids mutations on the A chain. The combination of recombinant technologies and post translational chemical modifications resulted in the discovery of long-lasting double chain relaxin analogues^11^. The recent identification of a single chain peptide derived from the relaxin B chain (B7-33) as a functional agonist of RXFP1 was an important step towards simplification of the relaxin structure^12^. We have recently identified new short single B chain modified peptides that are highly potent RXFP1 agonists^13^. These peptides resulted from stepwise modification of the relaxin B chain. Introduction of suitable mutations and shortening of the optimal peptide length led to RXFP1 agonists with nanomolar potency and improved stability in physiological fluids. Modification of the C-terminal amino acid with a spacer and fatty acids, resulted in short single chain RXFP1 peptide agonists displaying sub-nanomolar activity, high subcutaneous bioavailability, with extended half-lives compatible with daily subcutaneous administration in patients. The objective of the present study was to further characterize this new class of RXFP1 agonists by demonstrating the ability of the compound SA10SC-RLX to promote RXFP1 target engagement both in vitro and in vivo in various translational experimental settings.

## Results

### Characterization of SA10SC-RLX as a selective RXFP1 agonist

In our efforts to discover long-lasting single chain RXFP1 agonists, the peptide analog SA10SC-RLX (Supplementary Figure S1) was identified which incorporated a spacer and fatty acid chains to promote its binding to albumin and increase its half-life^13^. In vitro and in vivo assays relevant to RXFP1 activation were then used to characterise the pharmacological profile of this compound.

**Figure 1:**
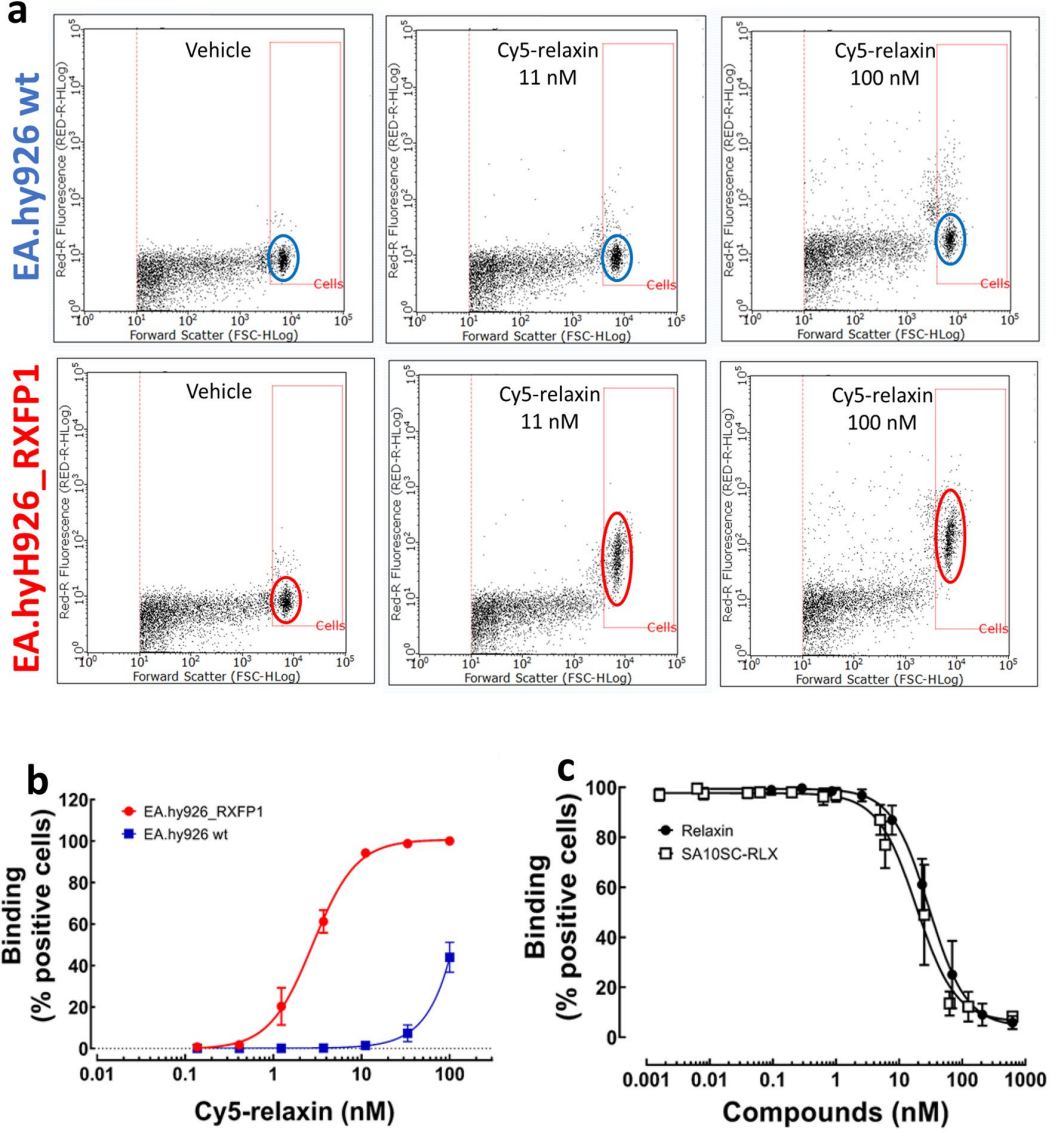
SA10SC-RLX binding to human RXFP1. Flow cytometry analysis (**a**) of two concentrations (11 and 100 nM) of Cy5-relaxin binding in wild type (wt) EA.hy926 cells (upper panel) or EA.hy926_RXFP1 cells (lower panel). The Y axis represents fluorescence intensity and the X axis the size of the particles. (**a**) is representative of 3 independent experiments with similar results. Concentration-dependent binding of Cy5-relaxin binding in wild type EA.hy926 cells (blue curve) or EA.hy926_RXFP1 cells (red curve) (mean ± SEM, n = 3 different experiments performed in duplicate) (**b**). Inhibition of 11 nM Cy5 relaxin binding by increasing concentration of unlabeled relaxin or SA10SC-RLX (mean ± SEM, n = 4 different experiments performed in duplicate) (**c**).

Flow cytometry was used to select cells expressing a detectable amount of RXFP1 receptor at the cell surface. Cells overexpressing recombinant human RXFP1, endothelial EA.hy926 (EA.hy926_RXFP1) or HEK (HEK_RXFP1) cells or with endogenous RXFP1 expression [OVCAR5 (Ovarian Carcinoma cells)] were used. EA.hy926_RXFP1 cells were selected based on the amplitude and specificity of the signal upon binding Cy5-relaxin tested at increasing concentrations (Fig. 1a, b). The best specific/non-specific signal ratio was selected at 11 nM Cy5-relaxin for 50,000 cells. SA10SC-RLX was then tested in competition with Cy5-relaxin to determine its affinity for RXFP1 in comparison with unlabeled relaxin. IC_50_ values obtained in these conditions were similar for both peptides showing that SA10SC-RLX can bind RXFP1 with similar potency to relaxin in EA.hy926-RXFP1 cells (Fig. 1c).

**Figure 2:**
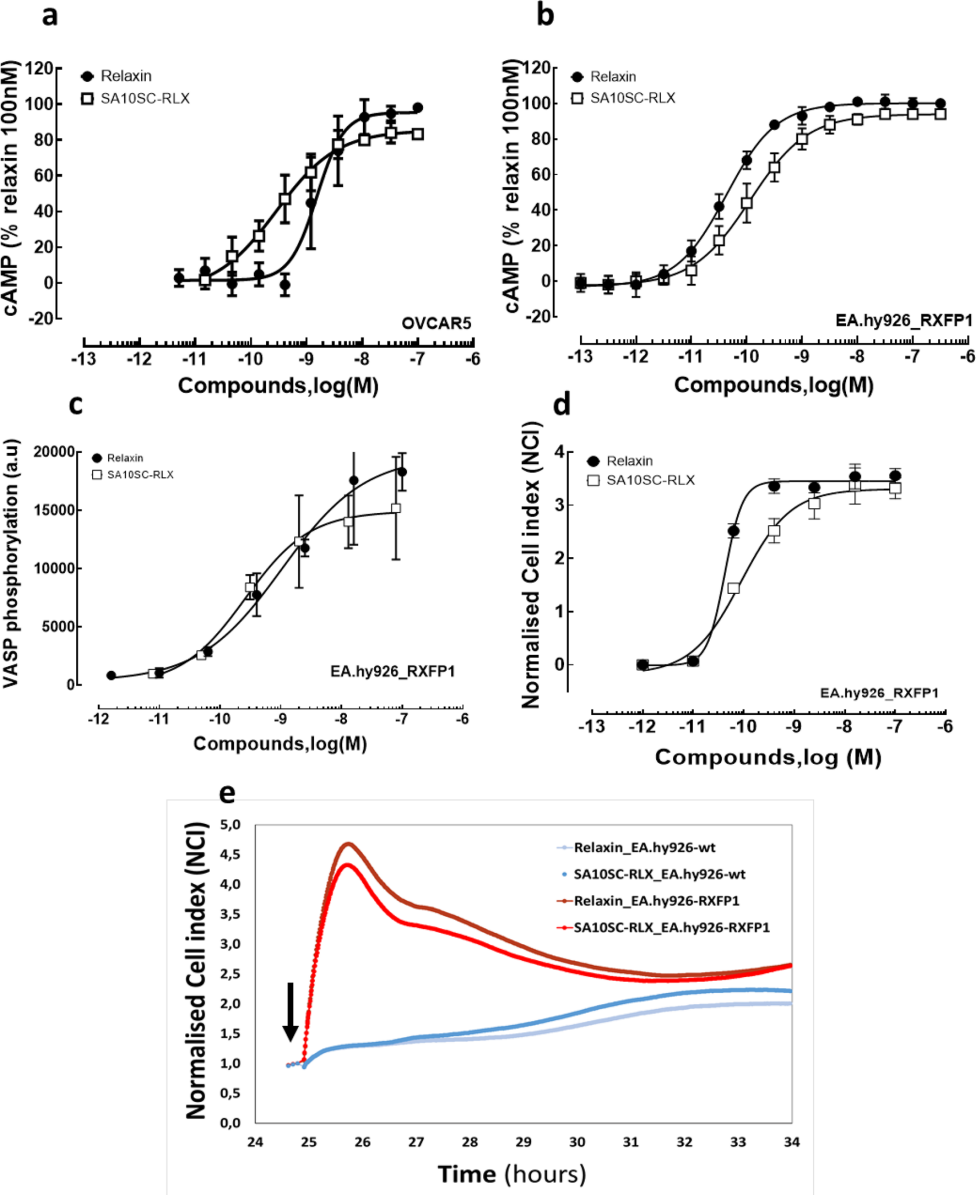
SA10SC-RLX and RXFP1 signaling. Concentration-dependent increases in cAMP induced by relaxin or SA10SC-RLX in human OVCAR5 cells expressing endogenous RXFP1 (**a**) (mean ± SEM, n = 6 different experiments performed in triplicate) or in EA.hy926_RXFP1 cells (**b**) (mean ± SEM, n = 5 different experiments performed in triplicate). Concentration dependent increases in VASP phosphorylation induced by relaxin or SA10SC-RLX or EA.hy926_RXFP1 cells (one representative experiment performed in duplicate out of 3) (**c**). Concentration dependent RXFP1 activation by relaxin or SA10SC-RLX recorded by real time impedance measurements (Xcelligence, CNI) in EA.hy926_RXFP1 (one representative experiment performed in duplicate out of 3) (**d**). Real time impedance trace over a period of 10 h after treatment with 10 nM relaxin or 10 nM SA10SC-RLX in wt EA.hy926 cells (blue lines) or EA.hy926_RXFP1 (red and orange lines) (one representative trace out of 3 different experiments) The arrow indicates the injection of the compounds (**e**).

RXFP1 is a Gs coupled receptor and the most robust response to relaxin treatment results in elevated intracellular cAMP concentrations^2^. RXFP1 activation was measured in OVCAR5 or EA.hy926_RXFP1 cells. Since ligand potency (EC50) determinations were performed consistently over a long period of time during compounds screening experiments, basal cAMP levels were heterogeneous from cell passage to passage. Data were thus determined and expressed as % of the 100 nM relaxin response performed as a positive control for each experiment. Relaxin and SA10SC-RLX increased cAMP in OVCAR5 or EA.hy926_RXFP1 cells with similar potency (Fig. 2a, b) and comparable results were also obtained in HEK cells overexpressing recombinant rat RXFP1 (Table 1).

**Table 1:** Potency and efficacy of SA10SC-RLX in head-to-head comparison with relaxin in different cell types with endogenous or recombinant expression of human or rat RXFP1. Relative EC50s (EC50rel) and Emax were determined with SAS procedure via Biost@t-SPEED-LTS v2.4 internal software using the 4-parameter logistic model. The geometric means of the EC50rel values were calculated with confidence intervals (CI) at 95% level. For Emax comparison between relaxin and SA10SC-RLX, normality hypothesis was confirmed for each group and a paired T test was performed. In the absence of normality hypothesis, a Wilcoxon test was performed. For the EC50 comparison, the same method was applied after log transformation of the value (*P < 0.05 vs relaxin).

Cell type/readout	OVCAR5 (n = 6)		EA.hy926_hRXFP1 (n = 5)		HEK_ratRXFP1 (n = 6)	
cAMP	EC50 (nM) [CI95%]	Emax (%) ± SEM	EC50 (nM) [CI 95%]	Emax (%) ± SEM	EC50 (nM) [CI 95%]	Emax (%) ± SEM
Relaxin	1.8 [0.9; 3.4]	96.2 ± 0.8	0.06 [0.04; 0.09]	100.1 ± 0.7	1.4 [0.9; 2.1]	95.6 ± 0.8
SA10-SC-RLX	0.3* [0.2; 0.7]	84.1 ± 1.1*	0.8* [0.3; 1.9]	93.7 ± 1.1	0.3* [0.1; 0.7]	68.5 ± 2.8*

Experiments were performed in EA.hy926_RXFP1 to measure the potency of relaxin and SA10SC-RLX on VASP phosphorylation, a downstream mediator of both the cAMP and cGMP pathways (via PKA and PKG). Since we found no evidence of NO production (with relaxin or SA10SC-RLX) in EA.hy926, we focused on VASP S157 phosphorylation as a marker of cAMP-dependent protein kinase (PKA), in contrast to S239 that is considered as marker of the cGMP-dependent protein kinase (PKG), Both relaxin and SA10SC-RLX induced an increase in VASP phosphorylation on S157 with potencies in the same range (EC50 [CI95%] relaxin: 0.18 nM [0.02;14] EC50 [CI95%] SA10SC-RLX: 0.19 nM [0.03;1.4]) as in the cAMP assays (n = 3) (Fig. 2c).

The differences in the engagement of some signaling pathway could be cell type dependent or due to the use of primary cells at very early passages. Since new RXFP1 agonists (B7-33, ML290) described so far are biased agonists^12,14^ impedance experiments were performed to assess the full phenotypic response to relaxin and SA10SC-RLX in EA.hy926 or EA.hy926_RXFP1 cells. Neither relaxin nor SA10SC-RLX induced a response in wild type EA.hy926 cells. In preliminary studies (n = 3), similar potency was observed on VASP phosphorylation for relaxin and SA10SC-RLX in EA.hy926_RXFP1 cells (Fig. 2d). When looking at the kinetics and the shape of the RXFP1 activation signal (Fig. 2e), superimposable responses were observed for relaxin (10 nM) and SA10SC-RLX (10 nM) over a period of 10 h in EA.hy926_RXFP1.

### SA10SC-RLX molecular interaction with RXFP1

Molecular modeling of the interaction of SA10SC-RLX with RXFP1 was performed to better understand its mode of interaction. The model of apo RXFP1 shows that each domain is stable after 200 ns in the molecular dynamics (MD) simulation, including the linker proposed to be in a helical conformation. In contrast to previous models where receptor domains were connected to one another without contacts^1^, the RXFP1 model becomes more compact after MD (Supplementary Figure S2a): the LRR domain tends to be in close contact with the membrane, rather than covering the extracellular side of the transmembrane domain. This opens a pocket suitable for relaxin binding on top of the transmembrane domain. The linker is in contact with the extracellular loops without specific interactions, bringing the LDLa module not far from the C-terminus of the LRR domain.

Relaxin and SA10SC-RLX were introduced into the MD simulations in order to identify a stable conformation that can be used as a starting point for MD in complex with RXFP1. The spatial organization of RXFP1 is not modified after relaxin binding (Supplementary figure S2b). The A-chain of relaxin is in contact with the extracellular loops of the transmembrane domain with a diffuse contribution of RXFP1 residues showing on the surface in agreement with previous studies^15^.

MD of SA10SC-RLX alone shows a stable helix for the Gly12-Ala21 fragment, the rest of the peptide is ordered by several internal Hydrogen bonds, for instance Glu23-Arg31^13^. The lipid moiety appears more disordered, but its globular shape is packed along the peptide and is maintained during MD, leaving the binding cassette totally free for binding to the RXFP1 LRR domain.

Global organization of RXFP1 allows a stable positioning of SA10SC-RLX (Supplementary Figure S3a) or relaxin (Supplementary Figure S3b) where the arginine cassette Arg13-XXX-Arg17-XX-Iso20 interacts with acidic residues in RXFP1 including D231, D279, E233 and E277 in agreement with experimental results obtained with relaxin^16^. The isoleucine of the arginine binding cassette is stuck against L204 and W182 for both ligands (Supplementary Figure S3a and S3b).

In addition to these common binding features, SA10SC-RLX shows an additional interaction involving its Trp 28. An original salt bridge involving Glu23-Arg31 blocks Trp28 in a conformation suitable for pi–pi interaction with Y80 in RXFP1^13^. This results in the alignment of aromatic residues involving Y80, F76, F72 in RXFP1 and ending with W68 strengthening the helical conformation of the linker (Supplementary Figure S3c). These results with SA10SC-RLX are different from relaxin where Trp28 does not unpack during dynamics preventing any specific interaction with the RXFP1 linker (Supplementary Figure S3d). This data is consistent with the lack of effect of mutations of Trp28 on the affinity of relaxin for RXFP1^17^.

**Figure 3:**
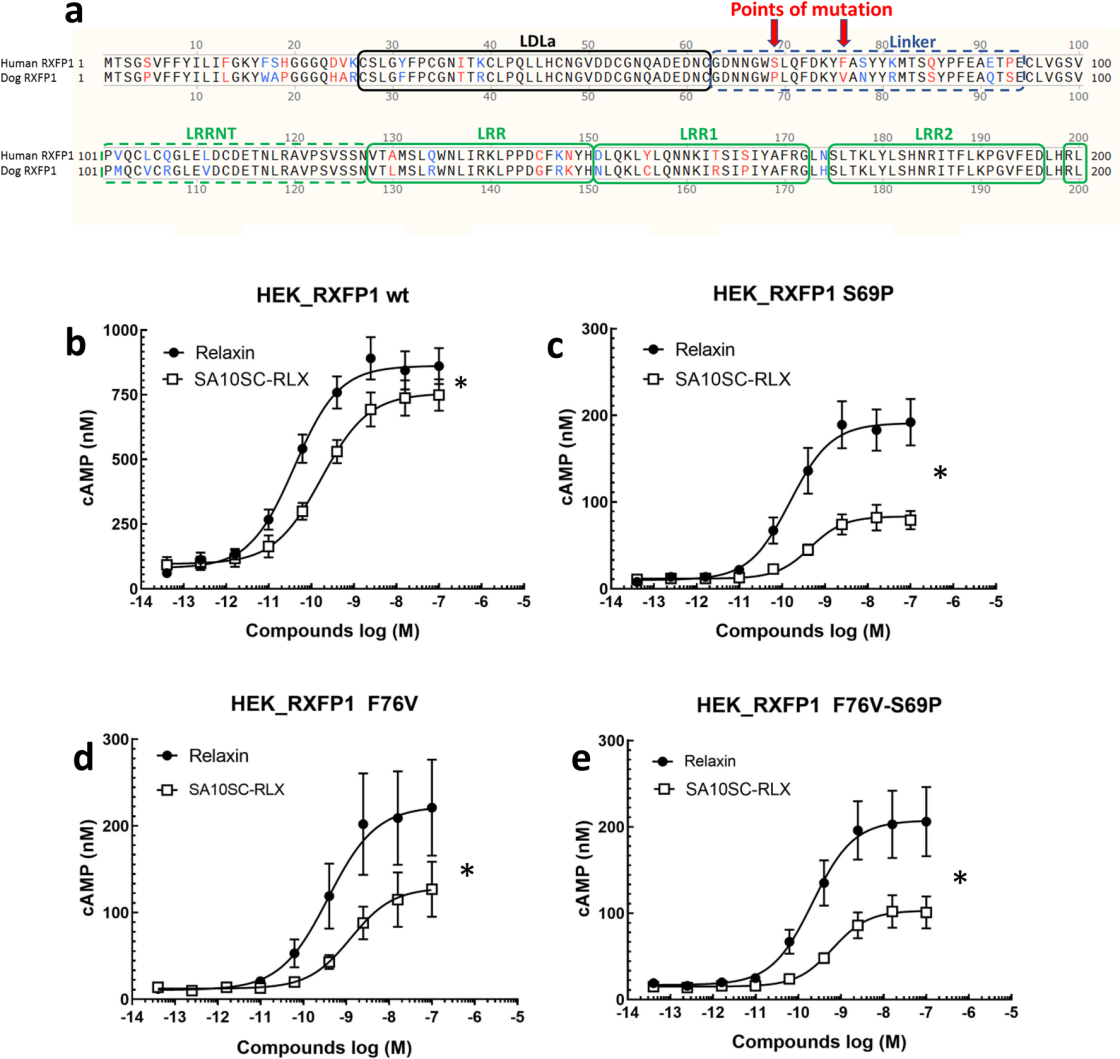
Role of linker domain in RXFP1 activation by SA10SC-RLX. Alignment of human RXFP1 (protein sequence NP_067647.2) with dog RXFP1 (sequence MW713050) across the first 200 amino acids of the N-terminus showing the LDLa and linker domains The arrows indicate the point mutations of interest (S69P and F76V) in the linker domain between the LDLa domain and the first LRR domain of the extracellular domain of RXFP1 (**a**). Concentration dependent effects of relaxin and SA10SC-RLX on cAMP production in HEK_RXFP1 cells (mean ± SEM, n = 4 experiments performed in duplicate) (**b**). Significantly decreased response of SA10SC-RLX compared to relaxin on cAMP production in HEK_RXFP1 cells mutated in the linker in position S69P (**c**), F76V RXFP1 (**d**) or F76V-S69P (**e**) (mean ± SEM, n = 4 experiments performed in duplicate). For Emax comparison between relaxin and SA10SC-RLX, normality hypothesis was confirmed for each group and a paired T test was performed. In the absence of normality hypothesis, a Wilcoxon test was performed (*P < 0.05).

To confirm the important role of the interaction of SA10SC-RLX with the RXFP1 linker domain, we extrapolated from data obtained in dog RXFP1. As we knew that SA10SC-RLX was poorly active on dog RXFP1 in contrast to relaxin (Supplementary Figure S4), we focused our attention on the specific dog (vs human) receptor mutations S69P and F76V in the linker of the LDLa globular domain (Fig. 3a). By analogy to our observations on the molecular models, we supposed that the two mutations contributed to modifying the environment allowing the interaction of SA10SC-RLX. S69P introduces a disruption in the helical conformation of the helix and breaks the alignment of aromatic residues limiting interaction with Trp28 in SA10SC-RLX. In addition, F76V removes the element in the middle of the aromatic alignment breaking the aromatic chain from SA10SC-RLX Trp28 to the Cys-Cys bridge in the LDLa domain. Therefore, S69P and F76V RXFP1 mutants were generated in parallel with the dual mutant S69P_F76V. cAMP was used as functional marker of the mutant receptors. As in other cell types, the activity was in the same range for H2 relaxin and SA10SC-RLX in HEK_RXFP1 cells (Fig. 3b). Relaxin activity (efficacy) was reduced in all RXFP1 mutants compared to the wild type construct which could be explain by lower expression of the receptor on the surface of the cells (Supplementary Figure S5). However, SA10SC-RLX activity was further reduced compared to relaxin (Fig. 3c,d,e). These results, in line with MD data, confirm that the interaction of SA10SC-RLX with the linker of the LDLa module of RXFP1 is key for its activity.

### SA10SC-RLX PK properties

**Figure 4:**
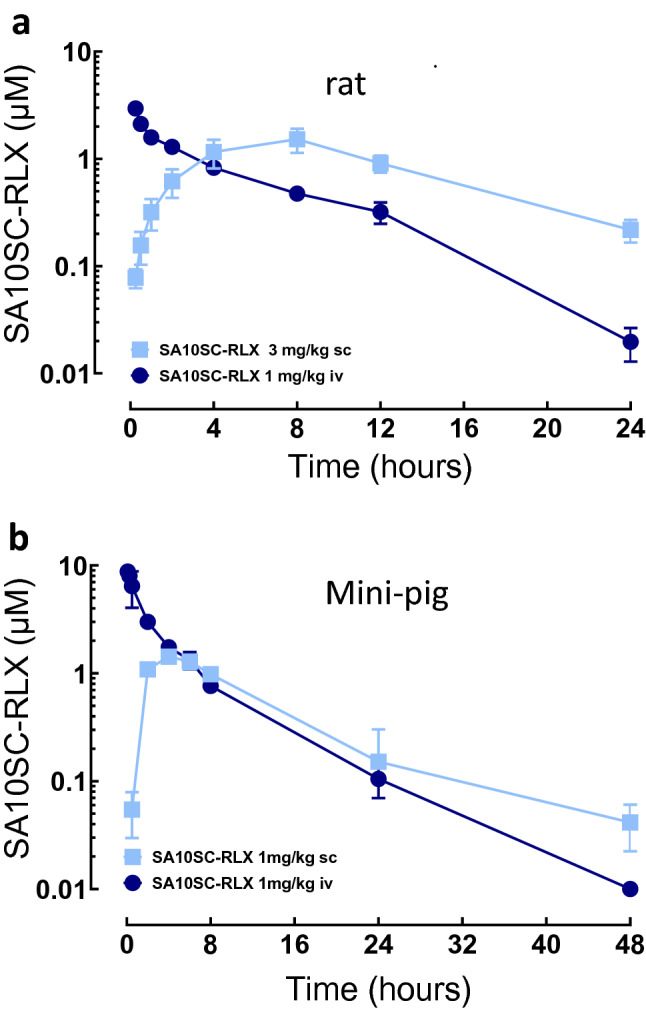
Pharmacokinetic properties of SA10SC-RLX after iv or sc administration in rats (**a**) or in minipigs (**b**) Blood samples were taken at each time points and SA10-SC-RLX was measured and quantified by LC-HRMS (rats) (mean ± sd, n = 3 animals per group) or LC-MS/MS (minipigs) (n = 2, individual data). Pharmacokinetics parameters were calculated by non-compartmental analysis.

Before being tested in translational models relevant to RXFP1 engagement, the pharmacokinetics of SA10SC-RLX was evaluated in Sprague–Dawley rats to support the pharmacological models and in Goettingen minipigs to assess its subcutaneous bioavailability. This species is better predictive of the subcutaneous PK profile in humans than other commonly used laboratory animals^18^. After intravenous administration of SA10SC-RLX at 1 mg/kg, the plasma clearance was considered as low at 0.43 mL/min/kg in rats and 0.14 ml/min/kg in minipigs with a long apparent terminal half-life of 4 h in rats and 7 h in minipigs. After subcutaneous administration of SA10SC-RLX at 3 mg/kg in rats or 1 mg/kg in minipigs, plasma concentrations were still in the 1 µM range after 12 h in both species (Fig. 4a, b). Following subcutaneous administration, the bioavailability calculated as the ratio of scAUC/ivAUC in minipigs was close to 70% (Supplementary Tables S1 to S4).

### SA10SC-RLX functional evaluation in rodent

**Figure 5:**
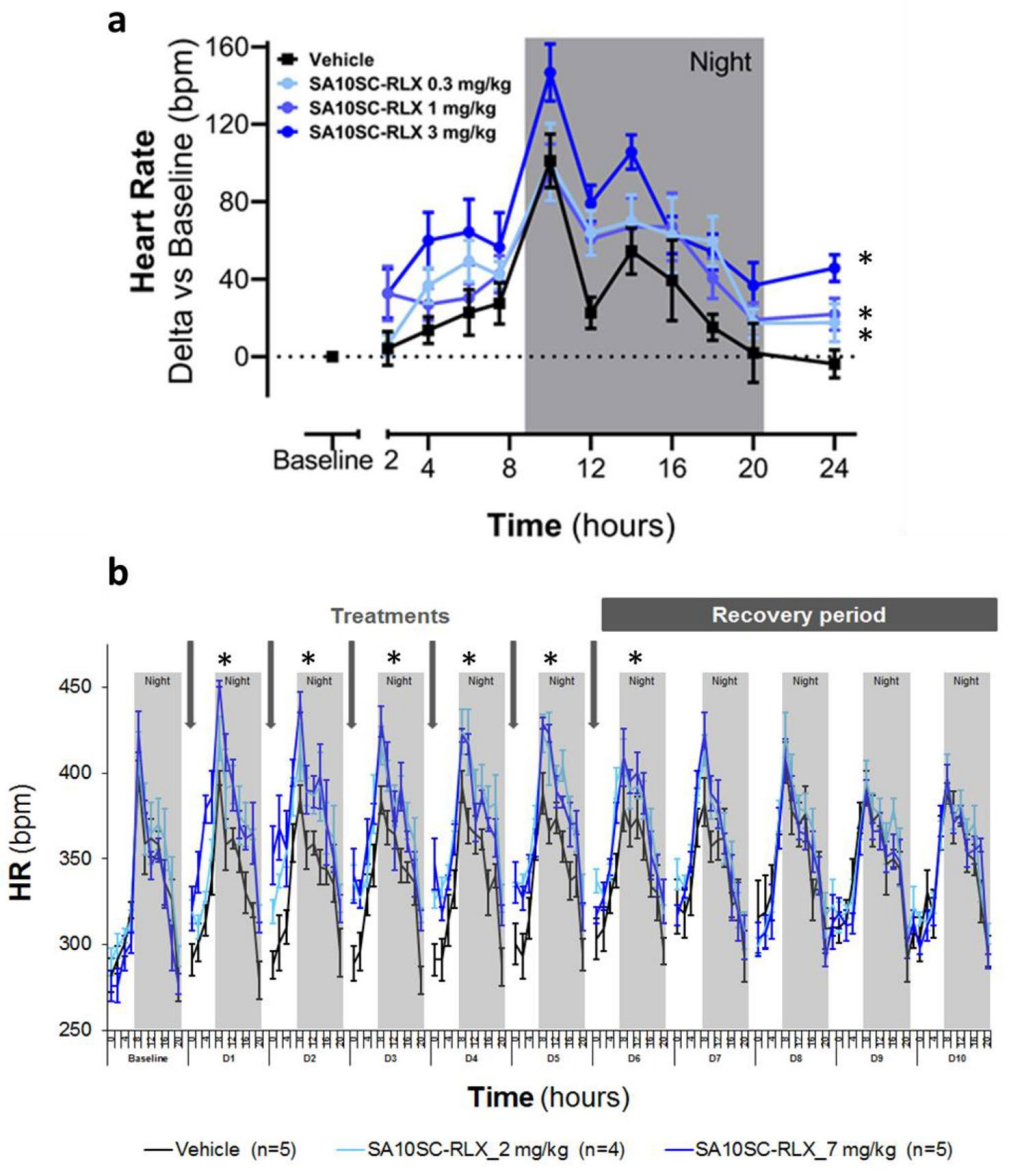
Effects of SA10SC-RLX in conscious healthy telemetered SD rats. Effects of a single SC administration of SA10SC-RLX at 3 doses (0.3, 1 and 3 mg/kg) on heart rate (HR) (changes from baseline) (**a**). Results are expressed as mean ± SEM (n = 8 animals/group). Whatever the dose considered, 0.3, 1 or 3 mg/kg, the mean effect of SA10SC-RLX is significantly different from the mean effect of the vehicle (p = 0.0001 at 0.3 mg/kg, p = 0.0032 at 1 mg/kg, p = 0.0002 at 3 mg/kg). Effect of repeated SC administrations of SA10SC-RLX at 2 doses (2 and 7 mg/kg) on HR (raw data, beat per minute (bpm)) (**b**). The arrows indicate the administration of the compound or the vehicle. Results are expressed as mean ± SEM (n = 5 animals/group) and are presented for each day (D1–D10 after baseline (24 h) recording for each animal). A two-way analysis of variance was performed on the raw data with fixed factors group and time, and if the group factor or the interaction group x time were significant, a global Dunnett’s test was carried out to compare the global effects of the treated groups to the global effect of the vehicle group (*P < 0.05).

Although not translational to humans, chronotropic effects following RXFP1 activation have been described in rodents with relaxin and small molecule agonists of RXFP1^19^. The effect of SA10SC-RLX on heart rate was therefore measured in conscious telemetered SD rats. A single subcutaneous administration with SA10SC-RLX at 0.1, 0.3 or 1 mg/kg led to a significant dose-dependent increase in heart rate (Fig. 5a) confirming RXFP1 activation in vivo. This effect was persistent over 15 h (Fig. 5a) in comparison to the relaxin effect that only lasted for 4–6 h (Supplementary Figure S6). SA10SC-RLX shows a longer half-life and activity in plasma than relaxin due to the binding of its fatty acid chain to albumin. However, this high albumin binding reduces its free fraction explaining why higher doses of SA10SC-RLX than relaxin are required in in vivo experiments.

To confirm the consistency of the pharmacodynamic effect of SA10SC-RLX after repeated subcutaneous daily administrations, the heart rate response was measured over a period of one week in conscious telemetered SD rats. SA10SC-RLX at 2 and 7 mg/kg/day led to a sustained and significant dose-dependent increase in heart rate that remained unchanged over 6 days of repeated SC injections, suggesting a lack of tachyphylaxis (Fig. 5b). A return to baseline was observed two days after the last administration, supporting a long-lasting RXFP1 engagement.

### SA10SC-RLX effect in a translational model of renal blood flow

**Figure 6:**
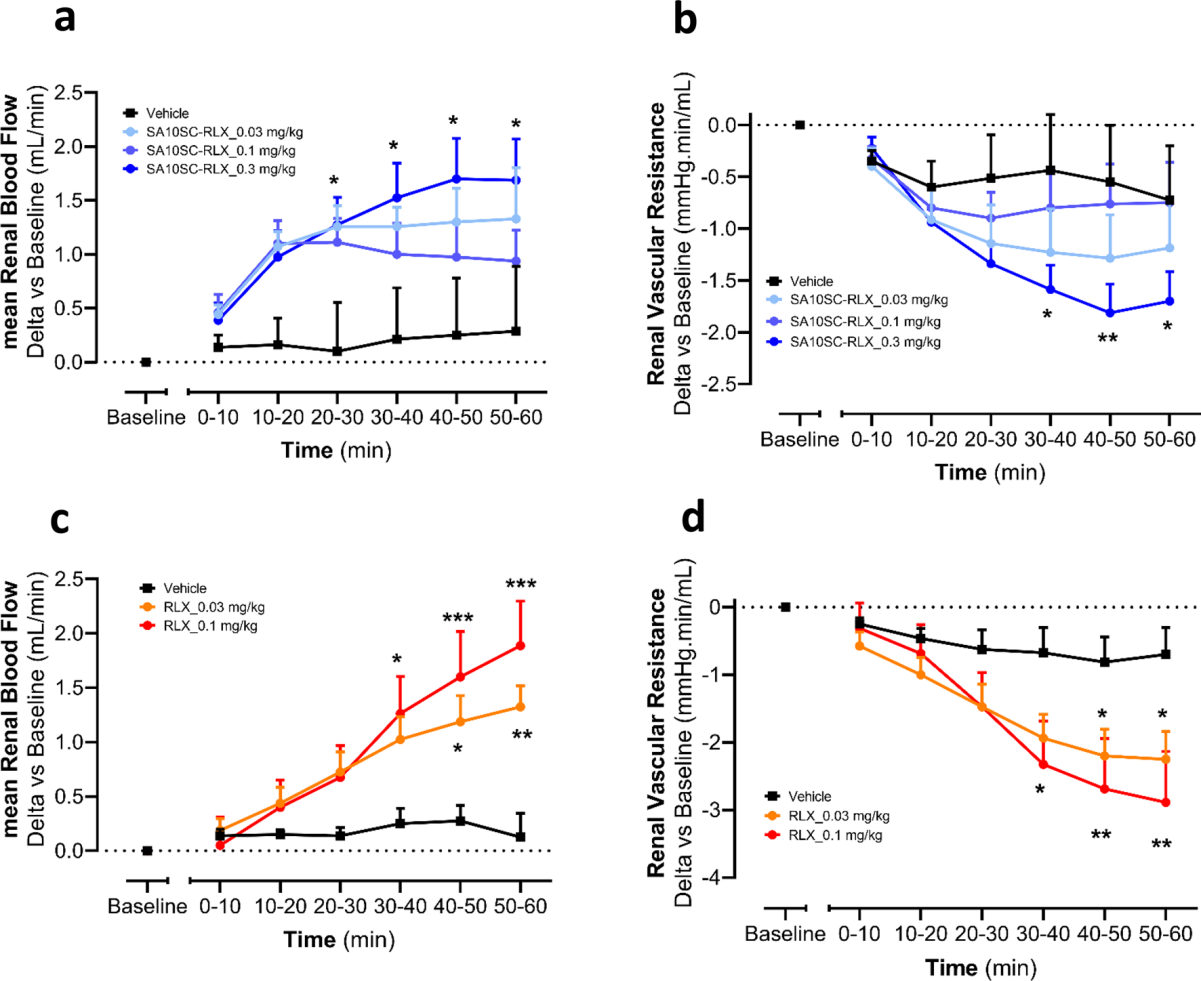
Effect of SA10SC-RLX and relaxin on renal blood flow and renal vascular resistance in anesthetized SD rat. Effects of IV administration of SA10SC-RLX (blue curves) or relaxin (orange and red curves) on mean Renal Blood Flow (RBF) (**a, c**) and renal vascular resistance (RVR) (**b**, **d**) in anesthetized SD rats. Results are expressed as mean ± SEM of changes from baseline for each animal (n = 8/group). A two-way analysis of variance was performed on rank-transformed data for both mean RBF and RVR, followed by two-tailed Dunnett's test, versus vehicle for group factor (*P < 0.05; **P < 0.01; ***P < 0.001).

In healthy subjects and heart failure patients, relaxin increases renal plasma flow (RPF)^20^. Thus, we compared the effect of SA10SC-RLX and relaxin on renal blood flow (RBF) in rats to generate translational data. SA10SC-RLX induced a dose-dependent increase in RBF (Fig. 6a) in parallel with a decrease in renal vascular resistance (RVR) (Fig. 6b) with no effects on blood pressure. A similar effect was observed with relaxin run in parallel experiments (Fig. 6c, d).

## Discussion

SA10SC-RLX belongs to a new class of long-lasting single chain peptides agonists of RXFP1^13^ which mimics relaxin activation both in vitro and in vivo in preclinical models.

Activation of RXFP1 is a complex multistep process. Previous studies have demonstrated that R13, R17 and I20, of the arginine cassette (RXXXRXXI/V, where x is any residue) of the H2 relaxin B-chain, bind to D231, D279, and E233, E277 located on LRR4–8 of the LRR domain of RXFP1^16^. However, ligand binding alone cannot activate the receptor and the LDLa module is essential for receptor activation^21,22^. Truncation or substitution of the LDLa module does not affect ligand binding but results in an inactive receptor^23^. The LDLa-LRR linker is intrinsically unstructured but a region of residual structure acts as a binding site for H2 relaxin, which in combination with the binding site of the LRR domain, is required for the nanomolar affinity of H2 relaxin to its receptor (Sethi et al. 2016). SA10SC-RLX binds to the arginine cassette in RXFP1 but has an additional interaction between its Trp 28 and Y80 in RXFP1^13^. This interaction allows the maintenance of the linker in a helical conformation ending with W68. Interestingly, during MD simulations, W68 comes into hydrophobic contact with the C47-C62 disulphide bridge of the LDLa module, inducing a bent conformation of the LDLa-linker assembly that could contribute to transmission of the activation signal. This kind of local arrangement of Cys-Cys/Trp is commonly observed in immunoglobulin domains^24^ or in transmembrane domains of GPCRs (W479/C485-C563 for RXFP1) but was not expected in a connector between two domains. These modeling results were supported by mutagenesis studies where alteration of the linker conformation with mutations limited the potency and efficacy of SA10SC-RLX. These results confirm the crucial role of the linker in the interaction with SA10SC-RLX to drive functional activation of RXFP1. These data in addition to molecular modeling propose a first vision of the mode of interaction of SA10SC-RLX with RXFP1 including the arginine cassette interaction in the peptide with the high affinity site in RXFP1 LRR and an additional interaction between Trp28 of SA10SC-RLX and Y80 in the linker domain of RXFP1.

SA10SC-RLX demonstrated high potency on human RXFP1 in different cell types and signaling pathways (cAMP/VASP) relevant to RXFP1 activation. Relaxin increases cellular cAMP in many cell types following RXFP1 activation coupled mainly with G_αs_ and adenylate cyclase activation^25^. SA10SC-RLX and relaxin increased cAMP production consistently over the different passages in cells expressing endogenous RXFP1 (OVCAR5) or recombinant RXFP1 (EA.hy926). The vasodilator-phosphorylated protein is a regulator of actin dynamics in endothelial cells and other cell types upon PKA activation^26^. VASP is regulated by phosphorylation, S157 being preferentially phosphorylated by the cAMP-dependent protein kinase (PKA), whereas S239 and T278 are targeted by the cGMP-dependent protein kinase (PKG)^27^. SA10SC-RLX was able to increase VASP S157 phosphorylation with similar potency to relaxin highlighting the importance of this cAMP pathway after RXFP1 activation in EA.hy926 cells. Beyond cAMP, RXFP1 activation is linked with other signaling pathways (NO, PI3K, ERK)^28^. In contrast to other groups^29^, we were not able to show activation of these alternative pathways in our experimental conditions in EA.hy926 cells. This discrepancy could be related to a difference in cell types used or in the number of passages used for the primary cells. However, impedance assays were performed as a phenotypic approach to study the global profile of RXFP1 activation in EA.hy926 cells. The profile of relaxin and SA10SC-RLX was superimposable showing that RXFP1 activation by both peptides leads to similar phenotypic cell response.

Beyond its potency in in vitro assays, SA10SC-RLX mimicked relaxin activity in pharmacological models. Relaxin induces a chronotropic effect in rodents and other species (rabbit or sheep)^30^ but not in humans^31^. In rodents this effect is mediated by RXFP1 activating L-type channel calcium current in sinoatrial node cells^32^. This property has been used to determine the pharmacodynamic activity of small molecule agonists of RXFP1 in humanized mice^19^. Even if this effect does not translate to humans (no heart rate change reported with recombinant relaxin in clinical trials), this remains a good functional endpoint for RXFP1 agonists in rodent. In telemetered rat, SA10SC-RLX induced an increased in heart rate that was reproducible after repeated subcutaneous administrations. This suggests that SA10SC-RLX is suitable for long-term chronic subcutaneous treatment, but this is also the first direct demonstration in vivo that RXFP1 can be sustainably activated over time after repeated stimulations without desensitization.

No formal PK/PD analysis could be performed since it was not possible to collect plasma samples during the pharmacology experiments without introducing bias in the PD measurements (bias related to the stress of animals during blood collection) whether they were blood pressure, heart rate or renal blood flow. However, pharmacokinetics studies in the rat and pig, demonstrated that the long half-life of SA10SC-RLX is compatible with a once daily subcutaneous administration. The binding of the lipid moiety of SA10SC-RLX to albumin has been exploited for other long-acting peptides like liraglutide which targets the GLP-1 receptor and has demonstrated extended actions in obese and diabetic patients^33^.

Finally, relaxin also plays an important role in vascular and renal adaptations during gestation 34). The reno-vasodilatory properties of relaxin have been reported in preclinical models^35,36^ consistent with those described in healthy subjects or heart failure patients^20^. The effect of SA10SC-RLX on renal blood flow in rats is in line with the improvement of renal function by relaxin in patients with heart failure^37^. Since, deterioration of kidney function is an important prognostic factor for worsening heart failure, RXFP1 activation by restoring renal blood flow and kidney function is anticipated to be beneficial for the treatment of acute decompensated heart failure.

In conclusion, all of the properties outlined above make SA10SC-RLX a good mimic of relaxin activity on RXFP1 but with additional long-lasting properties suitable for daily use in the clinic. SA10SC-RLX could therefore be a good candidate for the chronic treatment of acute or chronic heart failure. The recent negative results of clinical trials in acute heart failure in general^38^ have prompted investigators to reconsider the duration of treatment in order to cover the vulnerable phase (1–2 months) following hospital discharge^39^. Recent studies suggest that starting treatment very early in this period, could benefit patients to avoid the risk of new decompensation and rehospitalization^40^. This perspective opens the door for the development of new therapeutic classes including long-lasting RXFP1 agonists for the treatment of AHF patients. In addition, this new class of long-lasting RXFP1 agonists could also benefit patients with fibrotic diseases based on numerous examples of the protective effect of RXFP1 activation in multiple preclinical models of fibrosis^41^ and the necessity for chronic treatment.

## Methods

In general, studies were designed to generate groups of equal size, using randomisation and blinded analysis. Statistical analysis was undertaken only for studies where each group size was at least n = 5. In vitro experiments were performed in duplicate, or triplicate and the n designating the number of different experiments or different primary culture (patients) tested where appropriate.

### In vitro and in silico studies

#### Materials and reagents

The human umbilical vein cell line, EA.hy926 was obtained from ATCC (#CRL-2922). The EA.hy926_RXFP1 cell line was established in our laboratory with EA.hy926 infected with a lentivirus expressing human RXFP1 (coding sequence NM_021634.3) and a resistance gene to puromycin. Puromycin was used to select positive clones, kept during duplication of cells and removed only for selective experiments. Expression of hRXFP1 at the cell surface was checked by flow cytometry. The OVCAR-5 cell line was established from ascites fluid from a non-treated patient with an advanced-stage ovarian tumor. It has recently been classified as originating from the gastrointestinal tract, not of ovarian origin. These cells were obtained from NIH and are part of the NCI-60 human cancer cell line screen. These cells were selected among other cell type because they express significant endogenous levels of RXFP1 (determined by RT-PCR) over several passages. The dog kidney, distal tubule cell line, MDCK.1 was obtained from ATCC (#CRL-2935). MDCK.1_dog RXFP1 cell line was established in our laboratory with MDCK.1 infected with a lentivirus expressing dog RXFP1 (coding sequence MW713050_) and a resistance gene to puromycin.

All culture medium and antibiotics DMEM-Glutamax I (# 31966)/FCS Heat Inactivated (#10500)/1% Antibiotic–Antimycotic (# 15240-062)/0.5 µg/mL Puromycin (# A-11138-03) were purchased from GIBCO. Versene is an EDTA solution (0.48 mM) from Fisher Scientific (ref 15040066) used as a gentle non-enzymatic cell dissociation reagent.

Human recombinant relaxin (B-29/A-24) was obtained from R&D (#6586-RB/CF) as a lyophilized reagent dissolved in H_2_O at 10 µM and stored at − 20 °C. Mono-Cy5 human relaxin was obtained from Phoenix peptide Inc (#FC-035-62), resuspended in ultrapure water + DMSO 10% (100 µL) at 10 µM stock stored at + 4 °C in dark vials. IBMX (SIGMA #I5879) stock solution was prepared in DMSO at 1 M and was diluted at 1 mM final concentration in test medium. cAMP HTRF assay kits were from CisBio (#Gs Dynamic kit-62AM6PEC). Phospho-VASP (Ser157) whole Cell Lysate Kit was obtained from Meso Scale Discovery (#K151FFD-2).

SA10SC-RLX was manufactured and supplied by Sanofi (Chilly-Mazarin, France) and its structure corresponds to peptide 64 previously published (Mallart et al. 2021) (Supplementary Figure 1).

### Culture conditions and cellular assays

EA.hy926 or EA.hy926_RXFP1 were grown in complete growth medium (DMEM + 10% Fetal Calf serum + 1% Penicillin/Streptomycin + puromycin 0.5 µg/mL) at 37 °C and 5% CO_2_ in a humidified incubator. When cells reached 80–90% confluency, cells were detached from plastic using Trypsin–EDTA and were plated in 96 or 384-well plates according to the test.

OVCAR5 cells were grown in complete medium (RPMI 1640 + 10% Fetal Calf serum + 1% Penicillin/Streptomycin) at 37 °C and 5% CO_2_ in a humidified incubator. OVCAR5 cells were detached with accutase for 5 min and were plated in 384-well plates at a density of 8000 cells/well.

### Competition Binding Assay

For competition binding assays EA.hy926 or EA.hy926_RXFP1 cells were harvested with Versene instead of Trypsin–EDTA and were plated in 96-well plates at a density of 100,000 cells/well. All experiments were performed at room temperature in the dark. Serial dilutions of compounds were prepared in 50µL of HBSS in clear 96-well round bottom plate. Negative (assay buffer, HBSS) and positive (Mono-Cy5 human relaxin 12 nM) control samples were included in the assay. After addition of 50µL of cell suspension (100,000 cells/well), plates were incubated for 30 min at room temperature. Then the cells were washed with 200µL of HBSS and the plates were centrifuged at 400 g for 5 min at room temperature. The supernatant was removed from the pelleted cells by flipping over the plate. The cell pellet was recovered gently by addition of 300 µL of HBSS. 150 µL of this cell suspension was transferred to a new 96-well plate containing 150 µL of assay buffer for immediate quantification of fluorescence with a Flow cytometer (Guava easyCyte 6HT-2L/Guava soft InCyte 3.3 software for analysis). Recording excitation and emission wavelengths were respectively 642 nm and 660 nm and 5000 events (% Cy5 positive cells) per sample were analyzed.

To determine Mono-Cy5 human relaxin inhibition the following formula was applied:

$\left( {\% {\text{C}} - \% {\text{B}}} \right)/\left( {\% {\text{P}} - \% {\text{B}}} \right) \times 100$where C represents the % positive cells with compound at each concentration, B the % positive cells with HBSS and P the % of positive cells with Mono-Cy5 human relaxin (12 nM) alone.

### cAMP assay

Cells were plated in low-volume 384-well plates at a density of 4000 cells/well for EA.hy926_RXFP1 cells or 8000 cells/well for OVCAR5 cells in a final volume of 5 µL. The plates were briefly centrifuged at 800 rpm to allow the cell suspension to move to the bottom of the wells and then incubated for 24 h at 37 °C in 5% CO_2_. 5µL of the diluted compounds were then transferred in 384-well plate containing 5µL of cells in test medium. The assay plates were incubated for 30 min at 37 °C under 5% CO_2_. After 30 min the reaction was stopped by addition of HTRF revelation reagents with a multidrop dispenser. The two reagents were 5 µL/well of Anti-cAMP-cryptate diluted 20-fold in conjugate and lysis buffer, and 5µL/well of cAMP-D2 diluted 20-fold in conjugate and lysis buffer.

After overnight incubation at room temperature, HTRF signal was read at 620 and 665 nm on Phera Star (BMG LAbtech) plate reader. The HTRF signal was calculated as follows: ratio HTRF = [(signal 665 nm)/(signal 620 nm)] × 10^4^. Since EC50 determination were performed consistently over a long period of time during compound screening experiments, basal cAMP levels may change from cell passage to passage. Data were thus determined and expressed as % of 100 nM relaxin performed as a positive control for each experiment.

### Phospho-VASP assay

These experiments were conducted according to the MSD protocol (K151FFD-2) and using a Sector 2400 from Mesoscale Discovery. In order to reduce the basal phosphorylation activity of VASP protein, EA.hy926_RXFP1 cells were stimulated with effector solutions in basal DMEM (i.e., without serum). Cells were plated in 96-well plate at a density of 35.000 cells/well overnight in a volume of 200µL of cell culture media. Prior to stimulation, growth medium was discarded, and the cells were washed one time with basal DMEM medium, before being incubated for 4 h with basal DMEM medium at 37 °C in 5% CO_2_. 50 µL of the effector standard solutions (relaxin or SA10SC-RLX) were dispensed in triplicate directly onto the cells. For control cells, 50µL of basal DMEM medium without effector standard solution were added onto the cells. The microplates were incubated for 20 min at 37 °C in 5% CO_2_. All the following steps were carried-out on ice. Medium was aspirated and the cells were directly lysed by addition of 50μL of complete lysis buffer/well (lysis buffer is provided in the pVASP assay kit and prepared according to MSD instructions). The microplates were placed for 60 min under orbital agitation. The cell lysates were collected in round bottom microplate then stored at − 80 °C until pVASP content determination according to MSD protocol.

### XCELLigence assay

Cell impedance assays in both wild type EA.hy926 and EA.hy926_RXFP1 cell lines were performed using a MP RCTA xCELLigence Analyzer (ACEA Biosciences) on E-Plates. 20,000 cells/well were seeded in E-plates in 200 µL of growth medium and left to settle for 30 min at room temperature. Plates were then placed into the xCELLigence RTCA MP Instrument under 5% CO2 at 37 °C and impedance was continuously measured every 15 min overnight. The next day, a 4-h serum starvation was performed using the same medium but with 0.1% FCS (impedance measurement every 5 min). Relaxin and SA-10SC-RLX peptides were added to the wells and cellular impedance was measured every 10 s for 4 h and every minute for 16 h.

Data analysis was performed using RTCA2.0 software according to ACEA recommendations. Raw impedance expressed as cell index was normalized for each well by dividing individual Cell Index values by baseline Cell index values obtained 5 min before peptide treatment in order to obtain Normalized Cell Index (NCI): mean NCI from 4 replicates for each condition are presented.

### Building of RXFP1 model

Molecular modelling was conducted using Schrodinger tools: Maestro, Macromodel, Prime and Desmond with OPLS3 force field (Schrödinger Release 2019–3: MacroModel, Schrödinger, LLC, New York, NY, 2019; Desmond Molecular Dynamics System, D. E. Shaw Research, New York, NY, 2020. Maestro-Desmond Interoperability Tools, Schrödinger, New York, NY, 2020). Some facilities related to homology modelling in Schrodinger tools were used: GPCR-specific alignment, insertion in membrane according to the OPLM database, system regeneration in Desmond for large complexes.

Human RXFP1 is constituted of three distinct domains that are independent from one another: the RXFP1 LDL module is available in the PDB (PDB code: 2JM4), in contrary to the rest of RXFP1, and has been used as is. The other domains have been rebuilt independently before reassembling the whole structure. For the Leucine Repeat Rich (LRR) domain, several templates were tested and the ligand domain NoGo receptor (PDB code: 1OZN) was selected as template. In order to have the best alignment, particularly for the extracellular loops, the GPCR domain was rebuilt as a chimera of two templates: beta1-adrenergic receptor and delta opioid receptor, PDB codes: 2VTA and 4N6H respectively. These two templates were selected according to their overall good sequence alignment and the alignment was refined manually, if necessary, to fit the reference alignments already published^42^ and obtain the best probability of alignment for each transmembrane helix. This model of the GPCR domain alone was positioned in a membrane using OPM database^42^ and submitted to 200 ns of molecular dynamic simulation. In agreement with previous work, it was hypothesized that the linker between the LDLa and the LRR domain is in a helical conformation and was rebuilt accordingly. All the independent domains were reconnected manually without contacts with the membrane embedded GPCR domain. The assembled RXFP1 receptor model inserted in a membrane was prepared for MD simulation: cubic water box with 12 Angstroms buffer, SPC solvent model with addition of 0.15 M NaCl. After standard relaxation protocols, the system was submitted to 1 μs MD with the following parameters: NPT ensemble, target temperature 298 K, 2 fs timestep.

### Molecular dynamics (MD) simulation of peptides

SA10SC-RLX has been modelled by homology with the B-chain of relaxin (PDB code: 2MV1) for the peptidic part^13^. The lipidic moiety has been built in several extended conformations, each model was submitted to standard MD: cubic water box with 12 Angstroms buffer, SPC solvent model with addition of 0.15 M NaCl, standard relaxation protocol followed by 200 ns MD simulation (NPT ensemble, target temperature 298 K, 2 fs timestep). MD trajectories have been analyzed to conclude that even though lipid moiety conformations are very different in all simulations, they show a common globular fold along the peptidic helix on the side opposite to residues involved in RXFP1 binding. Based on these results a representative conformation of SA10SC-RLX was selected.

Schrodinger facilities allowing to restart from an already equilibrated system have been used to build RXFP1 with relaxin or SA10SC-RLX, both placed in the system not far from their putative binding site but not in contact with RXFP1. These two systems were submitted to 1 μs MD simulations with the following parameters: NPT ensemble, target temperature 298 K, 2 fs timestep.

### Cloning of Dog RXFP1 mRNA sequence

In silico analysis of the dog genome build CanFam3.6 (GenBank released 09/2011, annotation 104) revealed gaps in the potential RXFP1 gene and predicted mRNAs with the absence of one critical exon. Comparative analysis with other Caniformia sequences evidenced the potential existence of the lacking exon and the need to obtain the full dog RXFP1 coding sequence. Total RNA was thus extracted from dog coronary arteries (Marsh, USA) using RNeasy Fibrous Mini Kit (Qiagen). SuperScript III (Invitrogen) was used for cDNA synthesis. Forward primer 5’-TCAGTTGCTCAGAAAGAAGGAA-3’ and Reverse primer 5’-ATTTTGTGGCATCCATTTCAT-3’ were designed with Primer 3 software to cover the full coding sequence. PCR reactions were performed with Phusion High-Fidelity DNA polymerase (ThermoFisher). PCR products were purified from agarose gel using NuccleoSpin Gel and PCR clean-up (Macherey–Nagel) and then tailed for T/A cloning with Taq DNA Polymerase (Invitrogen). Cloning was done using pcDNA 3.1/V5 Topo TA Expression Kit (Invitrogen) and One Shot TOP10 chemically Competent cells (Invitrogen). Positive clones were amplified and sequenced using a 3730 DNA analyzer (Applied Biosystems) and resulting sequences were assembled with Vector NTI (Invitrogen). The datasets generated and/or analyzed during the current study are available in the GenBank repository. The complete sequence is available under accession number MW713050.1 (Canis lupus familiaris relaxin/insulin-like family peptide receptor 1—Nucleotide—NCBI (nih.gov)).

### Functional assays with RXFP1 mutants

HEK cells were stably transduced with lentiviral constructs (Vectalys, MOI 20, batches rV2.1A1.5057 B, rV2.1A1.5056 B and rV2.1A1.5059 B) containing a bicistronic vectors encoding hRXFP1 (S69P) or hRXFP1 (F76V) or hRXFP1 (S69P/F76V) under the EF1 promoter followed by an internal ribosome entry site (IRES) elements to express the neomycin antibiotic resistance gene for selection with 500 μg/mL Geneticin (Invitrogen, #10131-019). Three days post transduction, HEK cells were grown in the presence of Genetecin. Positive individual clones were selected and amplified, and cAMP readout (nM) was used as described for other cell types at a cell density of 8000 cells/well.

### In vivo studies

Male Sprague Dawley (SD) rats or female Dahl Salt Sensitive females were purchased from Charles River Laboratories (Italy and US, respectively). These strains of rat were used based on previous publications demonstrating RXFP1 activation^44,45^. Before the studies, all animals were housed in an air-conditioned room (22 ± 2 °C) with food (sterilized #106 from Safe, France) and water (filtered to preserve husbandry sanitary status) freely available (3–4 rats/cage for all the studies, except for telemetric studies where 1 rat/cage) and maintained under a 12-h light/dark cycle (light on: 7:00 AM). The animals were acclimatized for six days in the laboratory facilities before starting any study.

Surgical studies were performed under aseptic conditions (sterilized instruments, sterile operating field, mask and sterile gloves for operator) using betadine for cutaneous disinfection before any incision. Rats were anesthetized with 5% isoflurane, which was then progressively lowered and maintained at 2% until the end of the surgery. Rats were placed on thermostatically controlled heating pads, and body temperature was maintained at 37 °C.

These studies were performed in accordance with the European Community Standards on the Care and Use of Laboratory Animals and approved by the IACUC of Sanofi R&D. Randomization was performed on weight and sex before initiation of treatment and equal number of animals were used for each treatment group. We acknowledge that sex difference is an important issue and since RXFP1 activation has been tested historically in female rodents we have validated also its activation in male rats (telemetry and RBF experiments). For obvious reason, female rats were used to study the effect on nipple length.

All animal care and experimental procedures were approved by the local Animal Ethics Committee (registered by the French authorities as #CEEA-24), authorized by the competent authority under the project license number APAFIS #3307. The use of animals complied with European Directive 2010/63/EU and all studies were conducted in an AAALAC International accredited facility (#1455). Animal studies are also reported in compliance with the ARRIVE guidelines and with the recommendations made by the British Journal of Pharmacology (Lilley et al., 2020).

### Materials and reagents for in vivo studies

SA10SC-RLX was dissolved in 50 mM tris buffer/25 mg/mL glycerol adjusted to pH = 7.4, except for plasma stability and rat pharmacokinetic studies which are described in the corresponding sections.

Recombinant relaxin was obtained from R&D and dissolved in 50 mM Tris buffer/25 mg/mL glycerol adjusted to pH = 7.4.

### Pharmacokinetics

Sprague Dawley rats (10–12 weeks old) obtained from Charles Rivers Laboratories (Como, Iltaly) were dosed with study peptide at 1 mg/kg IV and 3 mg/kg SC as a solution in PBS (0.2 mg/mL and 0.6 mg/mL respectively). Blood samples were collected from individual animals at the following time points: 0.083, 0.25, 0.5, 1, 3, 6, 8, 24, 48 and 72 h (IV route) and 0.25, 0.5, 1, 2, 4, 8, 12, 24, 48 and 72 h (SC route). All samples, collected into K3-EDTA tubes, were centrifuged and plasma was harvested and stored at − 80 °C until analysis. Two hundred μL of 0.1% formic acid in methanol containing 10 ng/mL warfarin as internal standard were added to 50 μL of standard, QC and PK study samples to obtain the protein precipitation. Each sample was vortexed and centrifuged, for 20 min, at 17,000 rcf at 4 °C. Ten microliters of the extracted plasma samples were injected onto the column. Chromatographic separation was performed on an Ultimate 3000 (Thermo Scientific) chromatography system using an XSelect Peptide CSH C18, (50 mm × 2.1 mm I.D., 2.5 μm, 130 Å; Waters Corporation) with a flow rate of 0.500 mL/min at 60 °C and a two-solvent linear gradient system. Solvents A (water) and B (acetonitrile) contained both 0.1% formic acid. Phase B was kept at 2% for 0.5 min linearly increased to 70% in 2.5 min, kept at 70% for 1.0 min and then decreased to the initial 2% in 0.1 min; the column was equilibrated back to the initial conditions for 2 min before the next run. The Ultimate 3000 was coupled to an Orbitrap QExactive™ mass spectrometer (Thermo Scientific) in positive mode. Quantitation was performed in full scan. The optimized source parameters were as follows: ion spray voltage 2.9 kV, sheath gas flow 50 a.u., aux gas flow 15 a.u., aux T 300 °C, capillary T 320 °C, S-lens RF level 50%. Full MS parameters were as follows: mass range 150–2000, AGC target 1 × 10^6^, max injection time 250 ms, resolution 70,000 (FWHM @ 200 m*/z*). Data processing was performed using Xcalibur 3.0 (Thermo Scientific). Ion current of the [M + 5H]5 + of the SA10SC-RLX was extracted with 5 ppm window for quantitation (814.47219 m/z). A calibration curve with 12 standard points was prepared freshly on the day of analysis by spiking appropriate amounts of an intermediate working standard solution into blank rat plasma and ranged from 1.22 to 20,000 ng/mL (0.30–4910 nM). Three QC samples, each at six concentration levels from 5.0 to 15,000 ng/mL (1.22–3690 nM), were used for all analyses. Calibration curves were prepared by plotting the analyte to internal standard peak area ratio versus concentration. Calibration standards were fit by linear regression with 1/x^2^ weighting. QC samples (n = 3) were used at six concentration levels. Accepted calibration curve contained minimum 7 concentration levels within ± 20% of nominal. Accepted QCs were within ± 25% of nominal. PK parameters were calculated by non-compartmental analysis using Watson LIMS 7.5.

Goettingen minipigs (Ellegaard Göttingen Minipigs A/S, Soroe Landevej 302, DK-4261 Dalmose, Denmark) were dosed with the study peptide at a dose of 1 mg/kg IV and SC routes (0.1 mg/mL). Blood samples were collected from individual animals at the following time points: 0.083, 0.25, 0.5, 2, 4, 6, 8, 24, 48, 72, 96 and 168 h (IV route) and 0.25, 0.5, 2, 4, 6, 8, 24, 48, 72, 96 and 168 h (SC route). All samples, collected into K3-EDTA tubes, were centrifuged and plasma was harvested and stored at − 80 °C until analysis. Three hundred μL of 1% tri-fluoro acetic acid in acetonitrile containing 10 ng/mL irbesartan as internal standard were added to 30 μL of standard, QC and PK study samples to precipitate the plasma proteins. Each sample was vortexed and centrifuged, for 5 min, at 4000 rpm and at 4 °C. Two hundred and fifty microliters of supernatant were collected and evaporated under nitrogen at 37 °C in LoBIND 96 well plates. 150 µL of water/acetonitrile (90/10, v/v) were added to dissolve the evaporates and 10 µL of the extracted plasma samples were injected onto the column. Chromatographic separation was performed on a Thermo Vanquish (Thermo Scientific) chromatography system using a UPLC HSS T3, (50 mm × 2.1 mm I.D., 1.8 μm, Waters Corporation) with a flow rate of 0.600 mL/min at 40 °C and a two-solvent linear gradient system. Solvents A (water) and B (acetonitrile) contained both 0.1% formic acid. Phase B was kept at 10% for 1.1 min linearly increased to 90% in 2.5 min, kept at 90% for 1.0 min and then decreased to the initial 10% in 0.5 min; the column was equilibrated back to the initial conditions for 1 min before the next run. The Vanquish was coupled to an Orbitrap QExactive™ mass spectrometer (Thermo Scientific) in positive mode. Quantitation was performed in full scan. The optimized source parameters were as follows: ion spray voltage 3.8 kV, sheath gas flow 70 a.u., aux gas flow 10 a.u., aux T 300 °C, capillary T 250 °C, S-lens RF level 60%. Full MS parameters were as follows: mass range 150–2000, AGC target 1 × 10^6^, max injection time 250 ms, resolution 70,000 (FWHM @ 200 m*/z*). Data processing was performed using Xcalibur 3.0 (Thermo Scientific). Ion current of the [M + 5H]5 + of the SA10SC-RLX was extracted with 5 ppm window for quantitation (814.47219 m/z). A calibration curve with 9 standard points was prepared freshly on the day of analysis by spiking appropriate amounts of an intermediate working standard solution into blank rat plasma and ranged from 10 to 4000 ng/mL (2.46–983 nM). Three QC samples, each at four concentration levels from 40.0 to 3200 ng/mL (7.86–786 nM), were used for all analyses. Calibration curves were prepared by plotting the analyte to internal standard peak area ratio versus concentration. Calibration standards were fit by linear regression with 1/x^2^ weighting. QC samples (n = 3) were used at six concentration levels. Accepted calibration curve contained minimum 7 concentration levels within ± 20% of nominal (± 25% at the Lower Limit of Quantitation). Accepted QCs were within ± 20% of nominal. PK parameters were calculated using a non-compartmental analysis with Phoenix WinNonLin version 6.4 software.

### Hemodynamics in Sprague Dawley (SD) rat

The study was performed in SD rats (12–16-week-old) previously implanted with a telemetry device from Data Sciences International™ (DSI, Saint-Paul, USA), and allowed to recover for a minimal period of 2 weeks before treatment with vehicle, SA10SC-RLX or relaxin. We used n = 8 rats per group for single SC study and n = 5 rat per group for repeated SC study. To perform these studies, we had 2 times 10 rats implanted with a telemetry device to finally select 8 or 5 rats with good quality and physiological hemodynamics’ signals (mean arterial pressure in an 90–110 mmHg range). The statistical test used considered the correlation between observation within animal.

Surgery conditions: pethidine was given in drinking water for 2 days prior surgery. The day of surgery a subcutaneous administration of central and local analgesics with anti-inflammatory drug (fentanyl, bupivacaine, lidocaine, carprofen) was performed with long acting terramycin antibiotherapy as well. Post-surgery, buprenorphine was administered followed by 2 daily administrations of carprofen and buprenorphine. A last administration of terramycin was finally done at day 3 post-surgery.

Animal selection (inclusion/exclusion) was done prior to the study, selecting rat with good quality and physiological hemodynamics’ signals (mean arterial pressure in an 90–110 mmHg range).

SA10SC-RLX was administered by subcutaneous injection at 0.3, 1 and 3 mg/kg and relaxin was administered iv at 1, 3 and 10 µg/kg. For this purpose, rats were anesthetized with inhaled 5% isoflurane for induction, intubated, and mechanically ventilated at 2% isoflurane. Animals were placed on controlled heating pads, and core temperature was maintained at 37 °C. The animals were equipped with DSI telemetry implants devices (HD-S10 or HD-S11 or HD S21) allowing the measurement of blood pressure (BP). BP was recorded through a catheter inserted into the abdominal aorta. At the end of the surgery, animals were placed in individual cages until the end of the study. The allocation of animals to each group was performed according to a cross-over design (Williams design). Hemodynamic parameters were recorded for 1 h before the treatment (basal period) and under continuous recording of the signal over a 24-h period (acute treatment study) or 10 days (repeated treatment study). Data acquisition was performed using Hem 4.3 acquisition software (Notocord®, Le Pecq, France) connected to DSI’s telemetry device.

Investigated parameters (calculated from BP signal) were Heart Rate (HR) in beats per minute (bpm), Diastolic and Systolic Blood Pressure in mmHg, Mean Blood Pressure in mmHg. Data were treated from Hem using Microsoft Excel®. For each parameter, a basal value was determined as the average calculated over a 1-h period during the pre-treatment sequence, and the values post treatment were calculated over 1–4-h periods.

### Renal blood Flow in SD rat

SA10SC-RLX, relaxin or vehicle was administered by IV route. SA10SC-RLX was administered at 0.03, 0.1 and 0.3 mg/kg and relaxin at 0.03 and 0.1 mg/kg.

SD Rats (10–12-week old) were anesthetized with inhaled isoflurane, placed on controlled heating pads, and core temperature was maintained at around 37 °C. Central and local analgesics with anti-inflammatory drugs (fentanyl, bupivacaine, lidocaine, carprofen) were administered prior surgery. Rats were then implanted successively with a jugular vein catheter for the administration of SA10SC-RLX, relaxin or vehicle, and a flow probe (Transonic® PRB 1 mm) around the left renal artery and an arterial blood pressure probe (SPR407, Mikro-Tip®, Millar Inc.) inserted into the abdominal aorta up to the left renal artery bifurcation via the femoral artery. Once implanted, after the stabilization of recorded parameters and a 10-min baseline period, rats received their treatment.

Parameters were then recorded for 1 h. Internal randomization software (Biost@t Rando V2.1) was used to allocate, based on body weight, either the 32 animals for SA10SC-RLX sub-study or the 24 animals for relaxin sub-study in one of the 4 treated groups (vehicle, SA10SC-RLX or relaxin at their three or two respective doses respectively i.e., 8 rats/group).

Investigated parameters were mean Renal blood Flow (RBF), Renal Vascular Resistance (RVR), total peripheral Resistance (TPR), Heart Rate (HR) and Mean Blood Pressure (MBP).

### Statistical methods

All data analysis and representation were performed according to the British Journal of Pharmacology guidelines for statistical analysis^46^. Group size is the number of independent values, and statistical analysis (n > 5) was done using these independent values (i.e., not treating technical replicates as independent values). When the number of repeated experiments (n) was < 5 data were referred as preliminary.

Relative EC50s (EC50rel) were determined with SAS procedure NLIN in SAS system release 9.4 under Unix via Biost@t-SPEED-LTS v2.4 internal software using the 4-parameter logistic model^47^. Y = A + [C/(1 + exp(− B*(log(X) − M)))]. The parameters A and (A + C) are the lower and upper asymptotes, B is the slope of the curve at the inflexion point, and the M parameter is the logarithm of the concentration estimated at the inflexion point. The geometric means of the EC50 rel values were calculated with confidence intervals (CI) at 95% levels. Geometric mean and its 95% CI were obtained using internal software Biost@t-SPEED-LTS v2. For Emax comparison between relaxin and SA10SC-RLX, normality hypothesis was confirmed for each group and a paired T test was performed. In the absence of normality hypothesis, a Wilcoxon test was performed. For the EC50 comparison after log transformation of the value, the same method was applied.

For in vivo studies, biostatistician performed sample size estimations for each model based on previous internal data. A suitable statistical test, for which significance is obtained at fixed difference of interest (in a range of 20–50%), power (usually 80%) and alpha risk (usually 0.05), which depends on the models and the readouts was used. ANOVA and post-hoc tests analyses were performed after hypothesis of normality and variance homogeneity were fulfilled.

Telemetry studies: A two-way analysis of variance was performed on the raw data with fixed factor group and time, and if the group factor or the interaction group x time were significant, a global Dunnett’s test was carried out to compare the global effects of the treated groups to the global effect of the vehicle group. The Dunnett’s test was also performed at each time point.

Renal blood flow studies: A two-way analysis of variance was performed on rank-transformed data for both mean RBF and RVR, followed by two-tailed Dunnett's test, versus vehicle for group factor.

A result was considered statically different when *P* < 0.05 and specific P value are indicated in graphs.

All animals included in the studies were kept for final analysis. Potential outliers were included in data analysis and presentation unless specified in results and/or data legend.

All statistical analyses were performed using SAS 9.4 (SAS Institute Inc., Cary, NC, USA) on Windows 7 PC.

## Supplementary Information

Supplementary Information.

## Data Availability

The dataset generated for the cloning of dog RXFP1 during the current study is available in the GenBank repository. The complete sequence is available under accession number MW713050.1 (Canis lupus familiaris relaxin/insulin-like family peptide receptor 1—Nucleotide—NCBI (nih.gov)). The other datasets generated during and/or analyzed during the current study are not publicly available due to potential conflict of interest with other competitors developing RXFP1 agonists but are available from the corresponding author on reasonable request.

## Abbreviations

RBF: Renal blood flow
MD: Molecular dynamic
NCI: Normalized cell index
CI: Confidence interval
wt: Wild type
HF: Heart failure
IV: Intravenous
SC: Sub-cutaneous
PK: Pharmacokinetic
SD: Sprague Dawley
sd: Standard deviation
AUC: Area Under the Curve
