# Supplementary material for: Characterization of a new potent and long-lasting single chain peptide agonist of RXFP1 in cells and in vivo translational models
Source: Sci Rep. 2022 Nov 28;12:20435. doi: 10.1038/s41598-022-24716-2 (PMC9705314; doi:10.1038/s41598-022-24716-2)
Supplement: Supplementary file 1 — Supplementary Information. [file 41598_2022_24716_MOESM1_ESM.pdf]

SUPPLEMENTARY MATERIAL

Characterization of a new potent and long-lasting single chain peptide agonist of RXFP1 in human cells and in vivo translational models

Stephane Illiano[†*], Bruno Poirier[†], Claire Minoletti[§], Olivier Pasquier [#], Laurence Riva[†], Xavier Chenede[†], Isabelle Menguy[§], Michel Guillotel[†], Philippe Prigent[†], Stéphane Le Claire [†], Florence Gillot[†], Gilbert Thill[$], François Lo Presti[†], Alain Corbier[†], Jean-Christophe Le Bail [†], Patrick Grailhe [†], Edith Monteagudo [‡], Raffaele Ingenito [‡], Elisabetta Bianchi [‡], Christophe Philippo[†], Olivier Duclos[§], Sergio Mallart[§], Ross Bathgate[¥], Philip Janiak [†].

† Cardio-Vascular and metabolism, Sanofi R&D, 1 avenue Pierre Brossolette 91385 Chilly Mazarin, France.

§ Integrated Drug Discovery, Sanofi R&D, 1 avenue Pierre Brossolette 91385 Chilly Mazarin, France

DMPK France, Sanofi R&D, 1 avenue Pierre Brossolette 91385 Chilly Mazarin, France

$ Translational Science, Sanofi R&D, 1 avenue Pierre Brossolette 91385 Chilly Mazarin, France

‡ Peptides and Small Molecules R&D Department, ≠DMPK, and Structural Biology IRBM Spa, Via Pontina Km 30 600, 00 071 Pomezia (ROME), Italy

¥ Florey Institute of Neuroscience and Mental Health and Department of Biochemistry and Pharmacology, The University of Melbourne, Parkville, VIC 3052, Australia

Correspondence and reprints request to:
*Stephane Illiano, PhD, IRSN, B.P. 17 – 92262 Fontenay-aux-Roses Cedex, France;

Email: stephane.illiano@irsn.fr, Tel: +33-1 58 35 80 71

Supplementary Table S1

Individual and Mean plasma PK parameters of SA10SC_RLX following IV administration at 1 mg/kg to male Sprague-Dawley Rats

Animal ID	Co (μM)	AUC (μM*h)	AUXext (%)	CL (mL/min/kg)	Vss (L/kg)	T1/2 (h)	number of PK points used for t1/2 calculation
1	3.02	8.48	1.01	0.48	0.14	4.16	5
2	3.36	9.32	1.01	0.44	0.11	3.49	5
3	3.28	10.9	1.02	0.38	0.12	4.11	5
Mean	**3.2**	**9.57**	**1.01**	**0.43**	**0.12**	**3.9**	
s.d.	**0.2**	**1**		**0.05**	**0.01**	**0.4**	

-

Supplementary Table S2

Individual and Mean plasma PK parameters of SA10SC_RLX following SC administration at 3 mg/kg to male Sprague-Dawley Rats

Animal ID	Cmax (μM)	Tmax (h)	AUC (μM*h)	AUC_{ext} (%)	F (%)
4	1.59	8	20.7	1.07	72
5	1.88	8	25.4	1.10	89
6	1.11	8	17.6	1.11	61
Mean	**1.5**	**8**	**21.2**	**1.09**	**74**
s.d.	**0.4**	**0**	**4**		**14**

Supplementary Table S3

Individual plasma PK parameters of SA10SC_RLX following IV administration at 1 mg/kg to female Göttingen minipig

**Animal ID.**	**C_0** (µM)	**AUC** (µM*h)	**AUC_{ext}** (%)	**CL** (mL/min/kg)	**V_{ss}** (L/kg)	**$t_{1/2}$** (h)	number of PK points used for t1/2 calculation
1	9.36	31.9	0.462	0.130	0.0439	7.06	5
2	8.57	27.1	0.479	0.151	0.0497	6.92	5

Supplementary Table S4

Individual plasma PK parameters of SA10SC_RLX following SC administration at 1 mg/kg to female Göttingen minipig

**Animal ID.**	**C_{max}** (µM)	**t_{max}** (h)	**AUC** (µM*h)	**AUC_{ext}** (%)	**F** (%)
3	1.42	6	23.8	3.16	81
4	1.45	4	16.6	1.83	56

Supplementary Figure S1

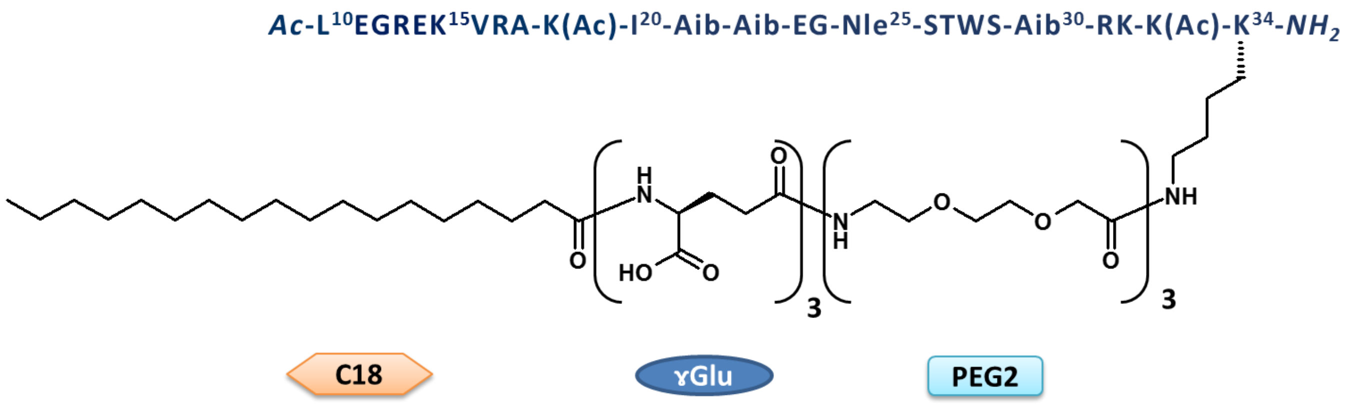

Supplementary Figure S1: **Structure of SA10SC-RLX.**
H2 relaxin B chain numbering has been chosen based on the first description of this series of peptides by Mallart et al. (2021). PEG2 was used as a spacer and Glu as linker to the lipid moiety (C18, CH_3-$(CH_2)_{16}$-CO-) used to promote albumin binding and increase peptide half-life.

Supplementary Figure S2

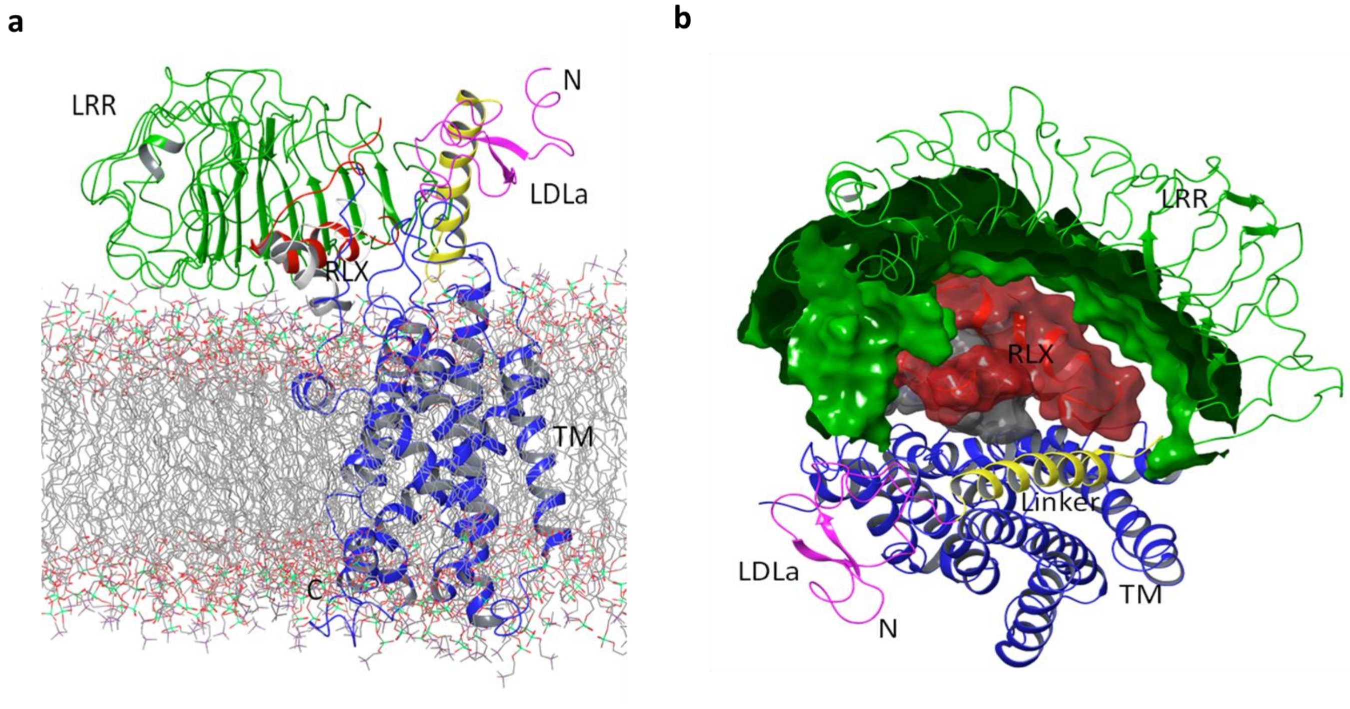

Supplementary Figure S2: Model of RXFP1 in complex with relaxin

Each domain is identified by a unique color: relaxin, A chain in grey and B chain in red. For RXFP1, LDLa is in magenta, the linker with LDLa in yellow, LRR in green and Transmembrane (TM) domains in blue. (**a**) is a side view of the model and (**b**) shows the contact surface view from extracellular side of relaxin and the LRR are shown, respectively in red and green to visualize the binding cassette.

<u>Supplementary Figure S3</u>

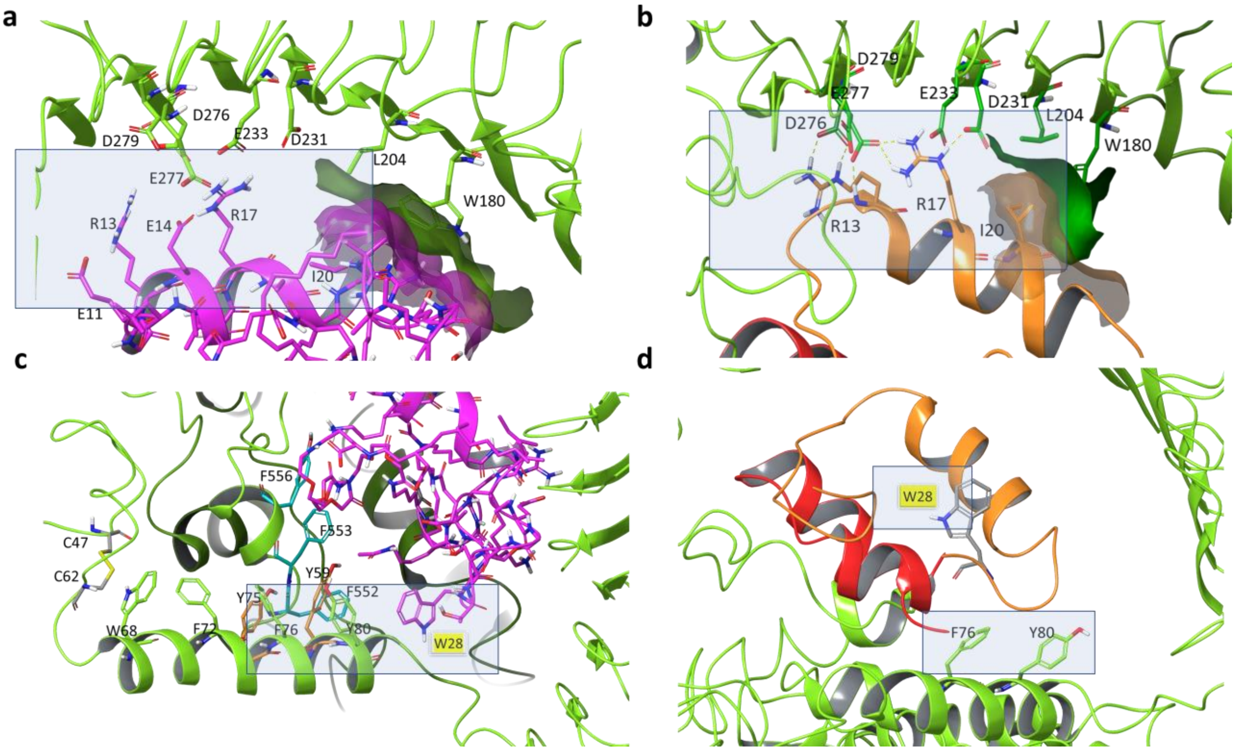

<u>Supplementary Figure S3</u>: Interaction network of SA10SC-RLX or relaxin with RXFP1. RXFP1 is in green, SA10SC-RLX in magenta and Relaxin in Orange (B Chain) and red (A chain). The grey boxes represent the specific domains of interaction

(a and b): Interaction with RXFP1 LRR domain

The three amino acids (R13-R17-I20) of the arginine binding cassette in SA10SC-RLX or relaxin are highlighted in grey boxes. The LLR is in green and SA10SC-RLX in magenta (**a**) and relaxin B chain in orange in (**b**). The contact surface between the peptides and LRR (green) is depicted respectively in orange and magenta for relaxin and SA10SC-RLX.

(c and d): Specific Interaction of W28 in SA10SC-RLX with the RXFP1 linker

Alignment of aromatic residues of the linker from W68 to Y80 (in green) interacting with W28 (yellow box) of SA10SC-RLX (in magenta). Aromatic residues of the linker (Y59, Y75, Y76) shown in orange are also in contact with aromatic residues (cyan, F552, F553, F558) of the RXFP1 TM domain. (**c**). In contrast, the relaxin positioning in the RXFP1 modeling shows that W28 in the relaxin B chain is not aligned with the aromatic residues of the RXFP1 linker (**d**).

Supplementary Figure S4

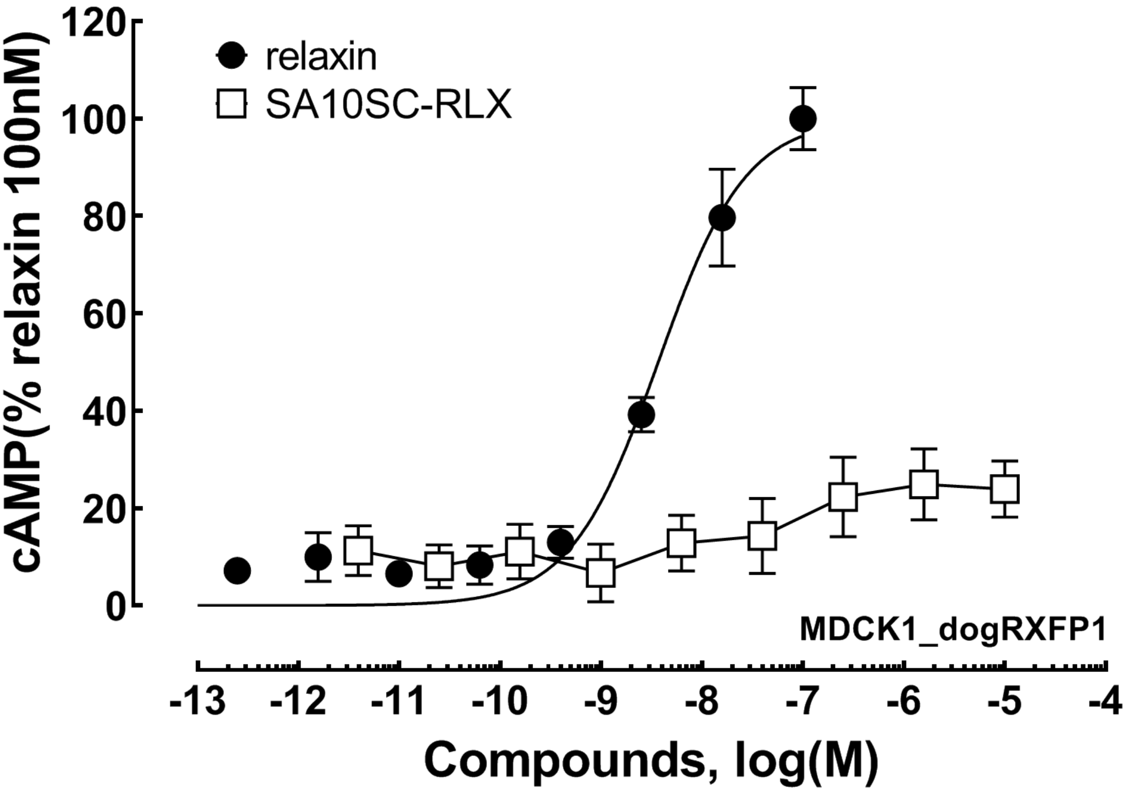

Supplementary Figure S4: Decreased response of SA10SC-RLX compared to relaxin on cAMP production in dog MDCK.1 cells with stable expression of recombinant dog RXFP1 (sequence id. MW713050) (mean +/- sem n=6 different experiments performed in duplicate).

Supplementary Figure S5

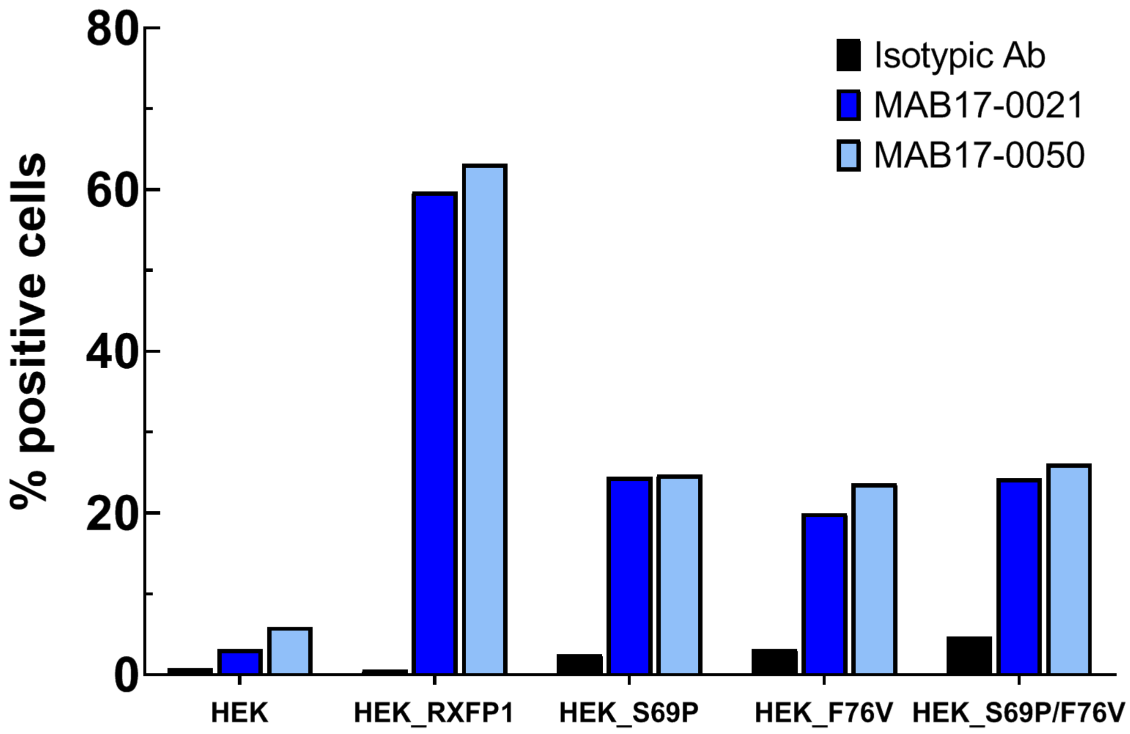

Supplementary Figure S5: Two different monoclonal anti-RXFP1 antibodies were obtained internally after immunization of rabbits with HEK_RXFP1 cells. The antibodies were utilized in FACS to detect RXFP1 and mutated RXFP1 at the surface of stable cell lines (HEK_RXFP1 / HEK_S69P /HEK_F76V or HEK_S69P/F76V). An isotypic antibody and wild type HEK cells (HEK) were used as negative controls.

Supplementary Table S5

Detection of RXFP1 and mutated RXFP1 at the surface of stable cell lines (HEK_RXFP1 / HEK_S69P /HEK_F76V or HEK_S69P/F76V) by FACS (Mean Fluorescence intensity for 3000 events/group). Same conditions as in Supplementary Figure S6.

	Ctrl Isotypic MAB + Goat anti-human-PE	MAB17-0021 + Goat anti-human-PE	MAB-0050 + Goat anti-human-PE
	Mean Fluorescence Intensity	Mean Fluorescence Intensity	Mean Fluorescence Intensity
HEK	18.1	20.9	24.0
HEK_hRXFP1 wt	14.9	225.2	276.1
HEK_hRXFP1 mut-S69P	22.6	48.6	57.3
HEK_hRXFP1 mut-F76V	20.9	43.0	51.4
HEK_HRXFP1 mut-S69P-F76V	23.8	49.1	55.5

Supplementary Figure S6

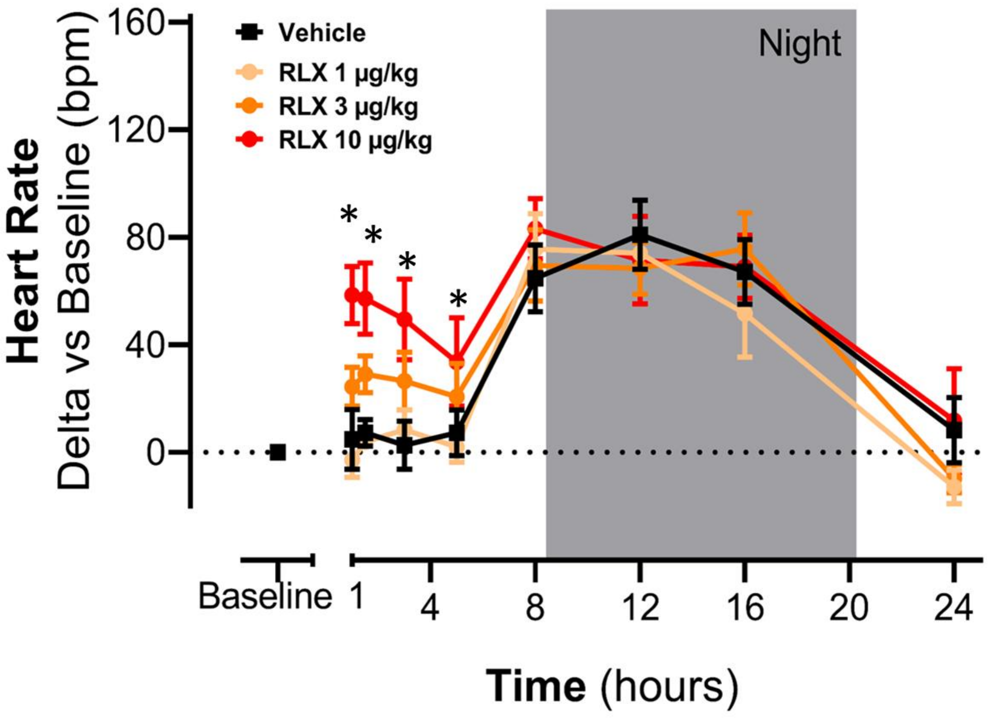

Supplementary Figure S6: Effects of a single iv administration of relaxin at 3 doses (1, 3 and 10 µg/kg) on HR in conscious healthy telemetered SD rats. Results are expressed as mean ± sem of changes from baseline for each animal (n=5/group). A significant difference (* P<0.05) between relaxin and vehicle was observed for the early time points (1-4h) at the doses of 3 and 10 µg/kg of relaxin.
